# GV21 enables ultrasound molecular imaging of tumor cell-associated VEGFR2 for early therapy assessment

**DOI:** 10.1016/j.mtbio.2026.103659

**Published:** 2026-09-08

**Authors:** Xiaoxin Liang, Shilin Lu, Liang Yang, Jianhua Zhou, Fei Yan

**Affiliations:** a Department of Ultrasound, State Key Laboratory of Oncology in South China, Guangdong Provincial Clinical Research Center for Cancer, Sun Yat-sen University Cancer Center, Guangzhou, 510060, China; b State Key Laboratory of Quantitative Synthetic Biology, Shenzhen Institute of Synthetic Biology, Shenzhen Institutes of Advanced Technology, Chinese Academy of Sciences, Shenzhen, 518055, China

**Keywords:** Ultrasound molecular imaging, Gas vesicles, VEGFR2, Tumor imaging, Therapy response

## Abstract

Vascular endothelial growth factor receptor 2 (VEGFR2) plays a crucial role in tumor growth through accelerating tumor angiogenesis. Recently, increasing evidence indicates that VEGFR2 is also overexpressed on tumor cells in multiple cancers, contributing to tumor progression and survival. BR55, a kind of VEGFR2-targeted microbubbles, has been developed and exhibited a promising potential for ultrasound molecular imaging (USMI) of early-stage tumors. However, it can only detect the VEGFR2 expressed on the tumor vascular endothelial cells but not on the tumor cells because it is strictly confined to the vasculature. Here, we developed VEGFR2-targeted gas vesicles (GV21) for USMI to enable detection of VEGFR2 in both vascular and extravascular interstitial regions. Unlike microscale BR55, GV21 has only 211.74 ± 1.05 nm particle size and can directly extravasate from tumor blood vessels to bind onto tumor cells, generating stronger and more persistent USMI signals in VEGFR2-expressing tumors. More importantly, the tumor-cell USMI signals can be specifically separated through subtracting the USMI signals of tumor blood vessels obtained from BR55-mimic microbubbles from the total USMI signals of tumor tissue from GV21 nanobubbles, allowing earlier and more comprehensive evaluation of therapeutic responses with the VEGFR2 tyrosine kinase inhibitor apatinib. These findings suggest that GV21 enables sensitive USMI of VEGFR2 expression and provides a promising approach for noninvasive evaluation of tumor molecular characteristics.

## 1 Introduction

Ultrasound molecular imaging (USMI) has emerged as a promising noninvasive modality for the dynamic visualization of molecular markers in vivo, owing to its advantages of real-time imaging, wide accessibility, and lack of ionizing radiation [1]. Targeted microbubbles have been widely used for USMI of tumor angiogenesis. Among them, BR55, a vascular endothelial growth factor receptor 2 (VEGFR2)-targeted microbubble, represents one of the most advanced clinically translatable agents [2]. Preclinical studies have demonstrated that BR55 enables sensitive and specific imaging of tumor angiogenesis across multiple tumor models, including pancreatic, breast, and prostate cancers [[3], [4], [5]], and enables early assessment of anti-angiogenic therapy response [[6], [7], [8]]. Importantly, BR55 has progressed into Phase 2 clinical trials and has shown feasibility for imaging of tumor vascularization in patients [9,10], highlighting its strong translational potential.

Despite these advances, an unavoidable limitation of these targeted microbubbles used in USMI such as BR55, is their relatively large particle size with typical diameters ranging from 1 to 10 μm [1], which strictly confines them to the vasculature and cannot extravasate into tumor interstitial spaces. Consequently, current microbubble-based USMI approaches primarily reflect molecular expression on vascular endothelium. VEGFR2, a key mediator of angiogenesis, has also been reported to be overexpressed in multiple tumor cell types and is associated with tumor aggressiveness and poor prognosis [[11], [12], [13], [14], [15]]. Therefore, BR55-based imaging strategies were restricted to the vasculature and might lead to incomplete characterization of VEGFR2 expression within the tumor microenvironment. Comprehensive assessment of VEGFR2 expression across both vascular and tumor cell compartments, along with therapy-induced microvascular changes, is essential for early evaluation of treatment response. However, current microbubble-based USMI remains challenging due to their confinement within blood vessels, limited vascular binding ability and reduced effective interaction with target sites [16,17].

**Fig. 1:**
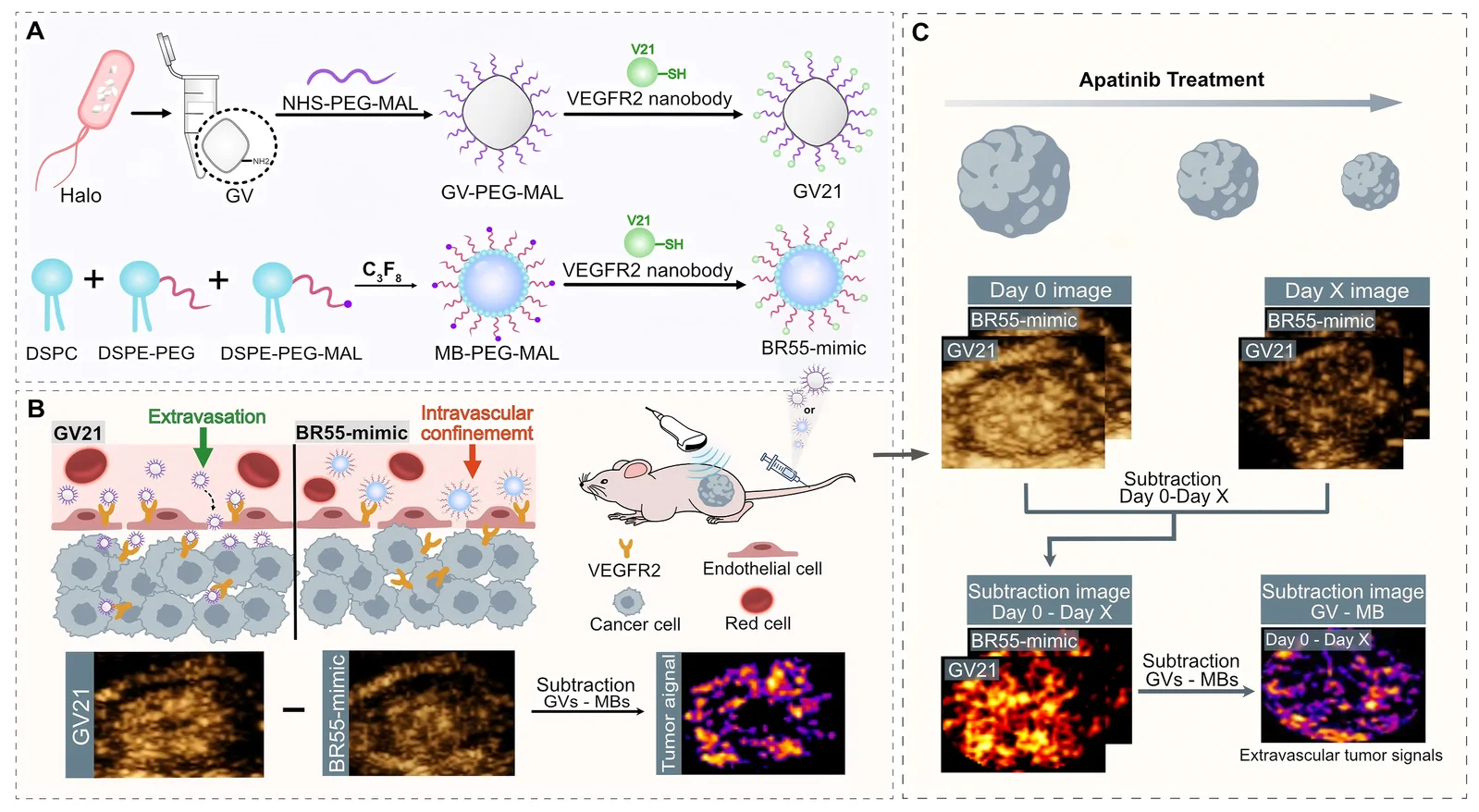
**Schematic illustration and ultrasound molecular imaging (USMI) of VEGFR2 using GV21 and BR55-mimic for therapy monitoring.** (A) Schematic representation of the preparation of VEGFR2-targeted gas vesicles (GV21) and VEGFR2-targeted microbubbles (BR55-mimic). (B) In vivo USMI in xenografted tumors derived from VEGFR2-positive tumor cells. GV21 enables VEGFR2 targeting in both vascular and extravascular tumor regions, whereas BR55-mimic primarily targets vascular VEGFR2 due to its intravascular localization. Subtraction of BR55-mimic signals from GV21 signals allows estimation of tumor cell-associated VEGFR2. (C) Therapeutic monitoring VEGFR2-targeted drugs with apatinib. Longitudinal USMI images were acquired at day 0 and subsequent time points (day X). Differences in GV21 and BR55-mimic signals between day 0 and day X were calculated, and signal subtraction of BR55-mimic from GV21 provided tumor cell-associated VEGFR2 expression during treatment.

Recently, biosynthetic gas vesicles (GVs) derived from *Halobacterium sp.* NRC-1 have attracted increasing attention as nanoscale ultrasound contrast agents. With a particle size of approximately 200 nm, GVs exhibit superior vascular extravasation and enhanced tumor-targeting capability. Previous studies have demonstrated that GVs provide robust contrast enhancement for liver and tumor imaging in mice using standard clinical ultrasound systems, highlighting their potential as promising nanocarriers for tumor USMI [[18], [19], [20]]. In this study, we developed VEGFR2-targeted gas vesicles (GV21) for USMI, which provides enhanced detection of VEGFR2 expression beyond the vascular compartment compared with VEGFR2-targeted microbubbles designed to BR55-mimic. This distinction enabled a subtraction-based strategy to separate vascular and tumor cell-associated VEGFR2 signals. The framework was further applied for longitudinal imaging of VEGFR2 dynamic characteristics during apatinib (a selective inhibitor of VEGFR2 tyrosine kinase) therapy [21]. By quantifying signal changes relative to baseline, this approach enables noninvasive assessment of treatment response and provides complementary insights into both vascular and tumor cell compartments. An overview of the study design is shown in Fig. 1.

## 2 Materials and methods

### 2.1 Materials

The anti-VEGFR2 nanobody (V21) was obtained from a previously reported literature [22] and expressed and purified in our laboratory. A GFP-binding nanobody reported previously [23] was used as a non-targeting control. Both nanobodies were engineered with a C-terminal 6×His tag and modified for site-specific conjugation via maleimide chemistry. Perfluoropropane (C_3_F_8_) was purchased from FluoroMed. The lipids, including 1,2-distearoyl-sn-glycero-3-phosphatidylcholine (DSPC), and 1,2- distearoyl-sn-glycero-3-phosphoethanolamine-N-[poly(ethylene glycol)-2000] (DSPE-PEG2000) were purchased from Avanti Polar Lipids. 1,2-distearoyl-sn-glycero-3-phosphoethanolamine-N-[maleimide (polyethylene glycol)-2000] (DSPE-PEG2000-MAL) and N-hydroxysuccinimide-polyethylene glycol 2000-maleimide (NHS-PEG2000-MAL) were purchased from RuixiBiotech.

### 2.2 Cell lines and animal models

All cell lines used in this article were purchased from the American Type Culture Collection (ATCC; Manassas, VA). The mouse brain microvascular endothelial cell bEnd.3, human lung cancer cell A549, human liver cancer cell line HepG2, human ovarian carcinoma cell line SKOV3, human pancreatic carcinoma cell line PANC-1, and human breast cancer cell line MDA-MB-231 were cultured in Dulbecco's Modified Eagle's Medium (DMEM, Gibco) supplemented with 10% fetal bovine serum (FBS, Gibco) and 1% penicillin-streptomycin (Gibco). Human colon cancer cell SW620, HCT116, RKO, LS174t and HT29 were cultured in RPMI 1640 supplemented with 10% FBS and 1% penicillin-streptomycin. Human umbilical vein endothelial cells (HUVECs) were cultured in endothelial growth medium-2 (EGM-2) supplemented with growth factors according to the manufacturer's instructions. All cells were cultured in a humidified incubator at 37 °C with 5% CO_2_. Female BALB/c nude mice (4-6 weeks old; 18-22 g) were purchased from Guangdong Medical Experimental Animal Center. Tumor xenografts were established by subcutaneous injection of 1 × 10^6^ SW620 or HT29 cells in 100 μL PBS into the right flank of each mouse. All animal experiments were approved by the Institutional Animal Care and Use Committee of the Shenzhen Institute of Advanced Technology, Chinese Academy of Sciences (protocol code: SIAT-IACUC- 211108-HCS-YF-A2079).

### 2.3 Preparation of Con-GVs and GV21

GVs were prepared from *Halobacterium sp.* NRC-1 (Halo) following a previously reported protocol [24]. Briefly, Halo was cultured at 37 °C with shaking (220 rpm) for 7 days. GVs were released with TMC lysis buffer (10 mM Tris-HCl, 2.5 mM MgCl_2_, 2 mM CaCl_2_, pH 7.5) and purified by repeated flotation centrifugation, followed by washing with PBS. The purified GVs were stored at 4 °C until use, and their concentration were initially measured using a microplate reader (Multiscan GO, Thermo Fisher, USA) at 500 nm (OD_500_). For the fabrication of GV21, the purified V21 nanobody was characterized by matrix-assisted laser desorption/ionization time-of-flight mass spectrometry (MALDI-TOF MS) using a microflex mass spectrometer (Bruker Daltonics, Germany). Subsequently, V21 nanobodies were covalently conjugated to the protein shells of GVs. Briefly, 1 mL of GVs (OD_500_ = 3.0) was first reacted with 8 mg NHS-PEG2000-MAL at 25 °C for 2h. After washing with PBS and centrifugation (250 g, 2 h), the obtained GVs-PEG2000-MAL from 1 mL of GVs (OD_500_ = 3.0) suspension were incubated with 50 μg V21 nanobody overnight at 4 °C. Finally, the resulting GV21 were collected by centrifugation and washed with PBS three times, and stored at 4 °C for future use. The concentration of purified GV21 was finally quantified using nanoparticle tracking analysis (NTA) with a ZetaView instrument (Particle Metrix). Control GVs (Con-GVs) were prepared using the same procedure with a non-targeting nanobody.

### 2.4 Preparation of Con-MBs and BR55-mimic

Lipid microbubbles were prepared using a DSPC:DSPE-PEG2000:DSPE-PEG2000-MAL molar ratio of 90:5:5. Phospholipids were dissolved in chloroform and dried under a nitrogen stream to form a thin lipid film. The film was hydrated and purged with perfluoropropane gas (C_3_F_8_, Flura, USA), then vibrated for 45 s to generate lipid-shelled microbubbles. Microbubbles were collected and washed three times by centrifugation at 400*g* for 3 min to remove excess lipids. Maleimide-functionalized microbubbles (5 × 10^8^ MB/mL, 1 mL) were subsequently incubated at room temperature for 1 h with 1 μg V21 nanobody to produce VEGFR2-targeted microbubbles (BR55-mimic), or with control nanobodies to produce control microbubbles (Con-MBs). Unbound nanobodies were removed by repeated flotation and washing. The microbubble concentration was finally calculated with an Automated Cell Counter (Thermo Fisher, USA).

### 2.5 Characterization and in vitro ultrasound imaging of MBs and GVs

Targeted and non-targeted control GVs (GV21 and Con-GVs) and microbubbles (BR55-mimic and Con-MBs) were characterized for physicochemical properties and imaging performance. Morphology and structure were examined by transmission electron microscopy (TEM; Hitachi H-7650, Hitachi Limited, Japan). Particle size and zeta potential were determined for all samples using a Zetasizer Nano S90 (Malvern Instruments, UK). To confirm successful nanobody conjugation, particles were incubated overnight at 4 °C with Alexa Fluor 647 (AF647)-labeled anti-6×His tag antibody (Abcam, UK), followed by washing three times with PBS and measurement of fluorescence intensity using a microplate reader. The conjugation efficiencies of GVs and MBs were estimated through a standardized calibration curve method.

To allow comparison between GVs and MBs in subsequent ultrasound imaging experiments, their concentrations were optimized to generate comparable signal intensities. For in vitro ultrasound imaging, particles at various concentrations were added in 1.5% (w/v) agar gel wells and imaged using a clinical ultrasound system (Resona 9, Mindray, China) equipped with an L11-3U linear array transducer in contrast mode. Imaging parameters were set as follows: acoustic power 2.57%, mechanical index 0.112, transmit frequency 7.1 MHz, and contrast gain 70 dB. Each experiment was repeated three times.

### 2.6 Western blot analysis of VEGFR2 expression in cell lines

HUVEC, bEnd.3, HepG2, RKO, SKOV3, HT29, A549, PANC-1, MDA-MB-231, SW620, HCT116, and LS174t cells were cultured in six-well plates for 24 h. Cells were washed with cold PBS and lysed in RIPA buffer (Sigma-Aldrich, Germany) for 30 min with occasional agitation. Lysates were centrifuged at 12,000 rpm for 15 min at 4 °C, and the supernatants were collected for protein quantification. Equal amounts of proteins were separated by SDS-PAGE and transferred onto PVDF membranes. Membranes were blocked with 5% non-fat milk for 1 h at room temperature and incubated overnight at 4 °C with anti-VEGFR2 antibodies (26415-1-AP, Proteintech, China) and β-actin (loading control), followed by HRP-conjugated secondary antibodies for 1 h at room temperature. Protein bands were visualized using enhanced chemiluminescence reagents (Thermo Fisher, USA) and imaged with tanon 5200 chemiluminescent imaging system.

### 2.7 Immunofluorescence staining and flow cytometry of VEGFR2 expression

SW620, HT29, and bEnd.3 cells (1 × 10^5^) were seeded on confocal dishes and cultured for 24 h. The cells were fixed in ice-cold acetone at −20 °C for 10 min and blocked with 5% bovine serum albumin (BSA) for 1 h. Following blocking, cells were incubated overnight at 4 °C with anti-VEGFR2 antibody (Thermo Fisher, USA), and then with AF647-conjugated secondary antibody (Thermo Fisher, USA) for 1 h at 25 °C. Finally, all cells were washed three times with PBS and stained with DAPI. Fluorescence images were acquired using a confocal laser scanning microscope (A1R, Nikon, Japan). For flow cytometry, SW620, HT29, and bEnd.3 cells (1 × 10^5^) were collected, stained with anti-VEGFR2 antibody for 1 h at 4 °C, and then AF647-conjugated secondary antibody for 45 min at 25 °C. After washed with PBS, the cells were analyzed by flow cytometry (BD FACSAria^TM^III, BD, USA). Control samples were treated with secondary antibody only.

### 2.8 Flow cytometric analysis of V21-binding cells

SW620, HT29, and bEnd.3 cells (1 × 10^5^) were collected and incubated with the V21 nanobody for 1 h at 4 °C. After washing, cells were stained with AF647-conjugated anti-6×His tag antibody for 45 min at 25 °C. The cells were then washed with PBS and analyzed using flow cytometry. Samples incubated with the secondary antibody alone served as negative controls.

### 2.9 In vitro cell binding ability of GV21 and BR55-mimic

SW620, HT29, and bEnd.3 cells (1 × 10^4^) were grown for 24 h in 48-well plates. Cells were fixed in ice-cold acetone at −20 °C for 10 min, washed in PBS for 3 times, and incubated darkly with 200 μL AF647-labeled particles at 1 × 10^7^ ⁻¹mL for 15 min at 25 °C. Unbound particles were removed by washing three times with PBS, and nuclei were stained with DAPI. Fluorescence images were acquired using an inverted fluorescence microscope (Ti2-E, Nikon, Japan). For competitive inhibition group, cells were pretreated with V21 antibody at 25 °C for 1 h to block VEGFR2 receptors before incubation with AF647-labeled particles.

### 2.10 Flow chamber experiments

1 × 10^4^ bEnd.3 cells were grown on single-chamber slide (ibidi, Germany) and cultured for 24 h. A syringe infusion pump (Wenhao, China) was used to pass solutions over the cells at flow rates corresponding to wall shear rates of 10, 50, and 150 s^−1^ [25]. The perfusion sequence was as follows: (a) PBS for 2 min, (b) 1 × 10^6^ particles of Con-MBs, BR55-mimic, Con-GVs, or GV21 in PBS for 4 min, and (c) a final 2-min PBS rinse. Cells were observed by phase-contrast microscopy (PCM, Olympus IX83 inverted microscope, Japan). Experiments were performed in triplicate for each kind of particles, and 10 randomly selected cells per slide were analyzed to quantify the number of bound particles per cell.

### 2.11 In vivo USMI

USMI was performed in SW620 and HT29 tumor-bearing mice (n = 5 per group) using a Resona 9 ultrasound system (Mindray) with a linear array transducer (L11-3U). Imaging was conducted 10 days after tumor inoculation. Tumor location and size were first identified by B-mode imaging, followed by contrast imaging with a mechanical index of 0.167, thermal index of 0.004, dynamic range of 115 dB, and contrast gain of 70 dB. Mice were anesthetized with 2% isoflurane and placed on a heating pad during imaging. Con-MBs (100 μL, 1 × 10^8^/mL), BR55-mimic (100 μL, 1 × 10^8^/mL), Con-GVs (100 μL, 1 × 10^12^/mL), and GV21 (100 μL, 1 × 10^12^/mL) were sequentially injected via the tail vein and imaged in the same tumor section. A minimum interval of 25 min was allowed between two injections, and a flash pulse was applied to remove residual contrast agents. Images were recorded for 10 min after each injection. Regions of interest (ROIs) were analyzed using ImageJ to obtain time-intensity curves. To estimate tumor cell-associated VEGFR2 signals, the BR55-mimic signals were subtracted from the GV21 signals within the same ROI.

### 2.12 Longitudinal USMI monitoring of apatinib treatment

To evaluate whether GV21 can monitor therapeutic responses in VEGFR2-positive tumor in addition to antiangiogenic effects, a longitudinal in vivo imaging study was conducted in mice bearing SW620 tumors. Tumor implantation was performed as described above, and treatment was initiated when tumors reached 100-150 mm^3^. Mice were randomly assigned to two groups (n = 5 per group): a control group receiving PBS and a treatment group receiving apatinib (50 mg/kg, 100 μL), administered by oral gavage once daily for 14 days. USMI was performed using both GV21 and BR55-mimic at baseline (day 0) and on days 1, 3, and 7 after treatment initiation. Tumor volumes and body weights were measured daily throughout the treatment period, and all mice were sacrificed on day 15 for endpoint analysis.

For quantitative analysis, ultrasound signals were measured in each tumor at 10 min post-injection for each time point (baseline, day 0; and days 1, 3, and 7), as described above. The changes in image signals relative to baseline were calculated for each time point (ΔGVs = GV21_Day0_ − GV21_DayX_; ΔMBs = BR55-mimic_Day0_ − BR55-mimic_DayX_). To specifically assess tumor cell-associated VEGFR2 signals, the ΔMBs signals were subtracted from the ΔGVs signals (ΔGVs–ΔMBs). These values were then used to evaluate changes in tumor-cell associated VEGFR2 signals over time. To further verify the robustness of the subtraction-based analysis, an alternative calculation method was also applied. Briefly, the signal difference between GV21 and BR55-mimic was first calculated at each time point (GV21 – BR55-mimic), and the changed signals relative to baseline were then determined (day 0 – day X). The results obtained using this method were compared with those derived from the ΔGVs − ΔMBs approach.

### 2.13 Histological examination

VEGFR2 expression and USMI correlation: Mice bearing SW620 or HT29 tumors (n = 5 per group) were sacrificed for histological analysis. Briefly, these tumors were collected and embedded in paraffin, followed by cutting and staining with immunolabeled anti-CD31 (Abcam, UK) and anti-VEGFR2 antibodies. Fluorescence images were captured using an upright microscope (Eclipse C1, Nikon, Japan). Mean fluorescence intensity (MFI) of VEGFR2 was quantified using ImageJ in the total tumor region, CD31-positive vascular regions, and CD31-negative tumor cell regions. GV21 signal, BR55-mimic signal, and the subtracted signal (GV21 − BR55-mimic) measured at 10 min post-injection were quantified within matched ROI. These imaging-derived signals were then plotted against VEGFR2 MFI from corresponding regions to assess correlations.

Assessment of extravasation: To examine whether GVs or MBs could extravasate through tumor endothelial gaps, SW620 and HT29 tumor-bearing mice were used. For each tumor model, mice were randomly assigned to receive AF647-labeled Con-GVs, GV21, Con-MBs, or BR55-mimic, with three mice per group. Each mouse received a single intravenous injection of 100 μL of the assigned contrast agents. Tumors were harvested 10 min post-injection and processed as frozen sections. Sections were fixed with 4% paraformaldehyde, incubated overnight at 4 °C with anti-mouse CD31 antibody, and subsequently stained with an AF488-conjugated secondary antibody (Abcam, UK) to visualize tumor vasculature. Nuclei were counterstained with DAPI, and fluorescence images were acquired using a confocal microscope. Representative images were selected from each group for analysis.

Temporal VEGFR2 and CD31 analysis: SW620 tumor-bearing mice were assigned to the treatment group or PBS control group. To establish baseline protein expression, three mice were sacrificed on day 0. Additional mice from each group were sacrificed at day 1, day 3, and day 7 post-treatment initiation (n = 3 per group per time point). Tumor tissue was sectioned for histological analysis. Paraffin-embedded sections were immunolabeled with anti-CD31 and anti-VEGFR2 antibodies. Fluorescence images were captured using an upright microscope, and representative fields were randomly selected for analysis.

Western blot analysis of VEGFR2 signaling: To further validate the early biological response to apatinib treatment, tumor tissues collected before and 24 h after apatinib treatment were subjected to western blot analysis as described above. The expression levels of VEGFR2 (2479, Cell Signaling Technology, USA), phosphorylated VEGFR2 (p-VEGFR2, 3770, Cell Signaling Technology, USA), p-AKT (AF0016, Affinity, USA) and β-actin were detected using specific antibodies.

### 2.14 Biosafety testing

Cell cytotoxicity: SW620 and bEnd.3 cells were seeded in 96-well plates (5 × 10^3^ cells/well) and incubated with GV21 or BR55-mimic at various concentrations for 24 h at 37 °C. Cell viability was then assessed using the Cell Counting Kit-8 (CCK-8) following the manufacturer's instructions, and absorbance was measured at 450 nm.

Hemolysis assay: Fresh blood from nude mice was collected via retro-orbital puncture, diluted with PBS, and then red blood cells (RBCs) were isolated by centrifugation (4500 rpm, 5 min). After five washes with PBS, RBCs were resuspended in 5 mL PBS and incubated with GV21, BR55-mimic at different concentrations, or dH_2_O (positive control) for 3 h at 37 °C. Samples were centrifuged (250 g, 2 h), and hemolysis was evaluated visually and quantitatively by measuring absorbance at 541 nm using a microplate reader.

In vivo biosafety: Nude mice (n = 3 per group) received an intravenous injection of 100 μL PBS, GV21 (10^12^ particles/mL), or BR55-mimic (10^8^ particles/mL). Serum was collected on days 1 and day 7 post-injection for blood biochemistry analysis (Wuhan Servicebio Technology Company, China). In a separate cohort (n = 3 per group), major organs (heart, liver, spleen, lung, kidney) were harvested 7 days post-injection and processed for H&E staining to assess tissue morphology.

### 2.15 Statistical analysis

GraphPad Prism 9.0 (GraphPad Software, San Diego, CA) was used for data visualization and statistical analysis. Data were expressed as mean ± standard deviation (SD). Statistical comparisons between two groups were performed using a two-tailed unpaired Student's t-test, whereas comparisons among multiple groups were performed using one-way analysis of variance (ANOVA). *P < 0.05, **P < 0.01, and ***P < 0.001 (two-sided) were considered indicative of a statistically significant difference.

## 3 Results

### 3.1 Preparation and characterization of GV21 and BR55-mimic

**Fig. 2:**
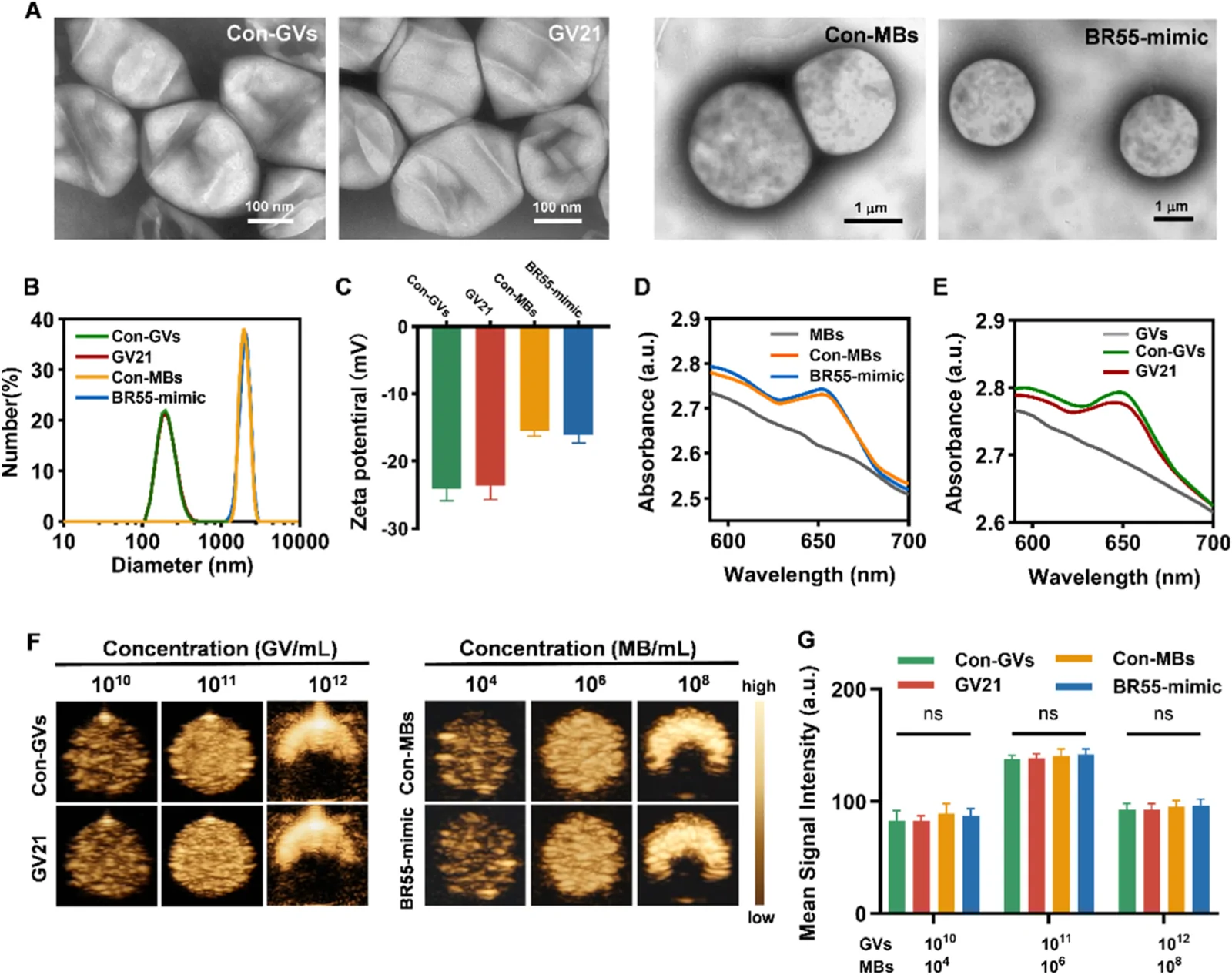
**Preparation and characterization of Con-GVs, GV21, Con-MBs, and BR55-mimic.** (A) TEM images of GVs (Con-GVs and GV21, scale bar = 100 nm) and MBs (Con-MBs and BR55-mimic, scale bar = 1 μm). Size distribution (B) and zeta potential (C) of GVs (Con-GVs and GV21) and MBs (Con-MBs and BR55-mimic). Absorbance spectra of MBs (Con-MBs and BR55-mimic) (D) and GVs (Con-GVs and GV21) (E). The representative ultrasound contrast images (F) and quantitative analysis (G) of GVs (Con-GVs and GV21) and MBs (Con-MBs and BR55-mimic) at different concentrations. Data of (B, C, and G) represent the mean ± SD from 3 independent experiments. (ns = nonsignificant).

The preparation of GV21 and BR55-mimic is illustrated in Fig. S1. The purified V21 nanobody exhibited the expected molecular mass as determined by MALDI-TOF mass spectrometry. As shown in Fig. S2, a predominant [M+H]^+^ peak was detected at *m*/*z* 15,598.56, consistent with the expected molecular mass of V21 (∼15 kDa). An additional peak at *m*/*z* 7795.38 was assigned to the doubly charged [M+2H]^2+^ ion of V21. The conjugation efficiencies of V21 to GVs and MBs were determined to be 38.68% and 43.75%, respectively. The resulting ligand density was estimated to be ∼800 V21 molecules per GV21 and ∼3.8 × 10^4^ V21 molecules per MB. Transmission electron microscopy (TEM) revealed that both Con-GVs and GV21 had spherical shapes with similar sizes (Fig. 2A, left). In contrast, Con-MBs and BR55-mimic displayed ball-shape structures with similar sizes (Fig. 2A, right). Dynamic light scattering analysis showed that the mean diameters of Con-GVs, GV21, Con-MBs, and BR55-mimic were 209.61 ± 2.62 nm, 211.74 ± 1.05 nm, 1986.40 ± 49.53 nm, and 2016.67 ± 132.17 nm, respectively (Fig. 2B). The corresponding zeta potentials were −24.05 ± 1.44 mV, −23.65 ± 1.65 mV, −15.50 ± 0.63 mV, and −16.12 ± 0.97 mV (Fig. 2C). No obvious differences in particle size or zeta potential were observed between control and GV21 or between Con-MBs and BR55-mimic. Successful conjugation of AF647-labeled V21 nanobody or AF647-labeled control nanobody to GVs and MBs was further confirmed by the characteristic absorption peak of AF647 at 648 nm (Fig. 2D and E). As shown in Fig. 2F and G, GVs at concentrations of 10^10^, 10^11^, and 10^12^ particles/mL produced ultrasound contrast signals comparable to those generated by MBs at 10^4^, 10^6^, and 10^8^ particles/mL, respectively (all *P* > 0.05). The higher concentrations required for GVs may be attributed to their much smaller particle size in comparison with MBs.

### 3.2 VEGFR2 expression and V21 nanobody binding in tumor cells

To investigate VEGFR2 expression in tumor cells, western blot analysis was performed in ten tumor cell lines, with bEnd.3 and HUVEC cells used as endothelial controls. As shown in Fig. S3, VEGFR2 protein could be detected in several tumor cell lines, including RKO, A549, PANC-1, MDA-MB-231, HCT116, and SW620, whereas HepG2, SKOV3, HT29, and LS174t exhibited minimal or undetectable VEGFR2 expression. Therefore, SW620 and HT29 cells were selected as VEGFR2-positive and VEGFR2-negative tumor cells for subsequent experiments, respectively. Immunofluorescence staining confirmed VEGFR2 expression in bEnd.3 and SW620 cells, whereas HT29 cells exhibited minimal VEGFR2 expression (Fig. S4A). Consistently, flow cytometry analysis revealed that 98.9% of bEnd.3 cells and 99.5% of SW620 cells were VEGFR2-positive, while only 3.45% of HT29 cells were positive for VEGFR2 (Fig. S4B).

To evaluate the cell specific binding ability of the V21 nanobody, flow cytometry was performed on bEnd.3, SW620, and HT29 cells. Fig. S5 showed that 97.9% of bEnd.3 cells and 80.9% of SW620 cells exhibited V21 binding, whereas minimal V21 binding was observed in HT29 cells (3.11%). Therefore, SW620 and HT29 cells can serve as VEGFR2-positive and VEGFR2-negative cell models for subsequent in vitro and in vivo studies, respectively.

### 3.3 Specific binding and flow-mediated adhesion of GV21 and BR55-mimic

**Fig. 3:**
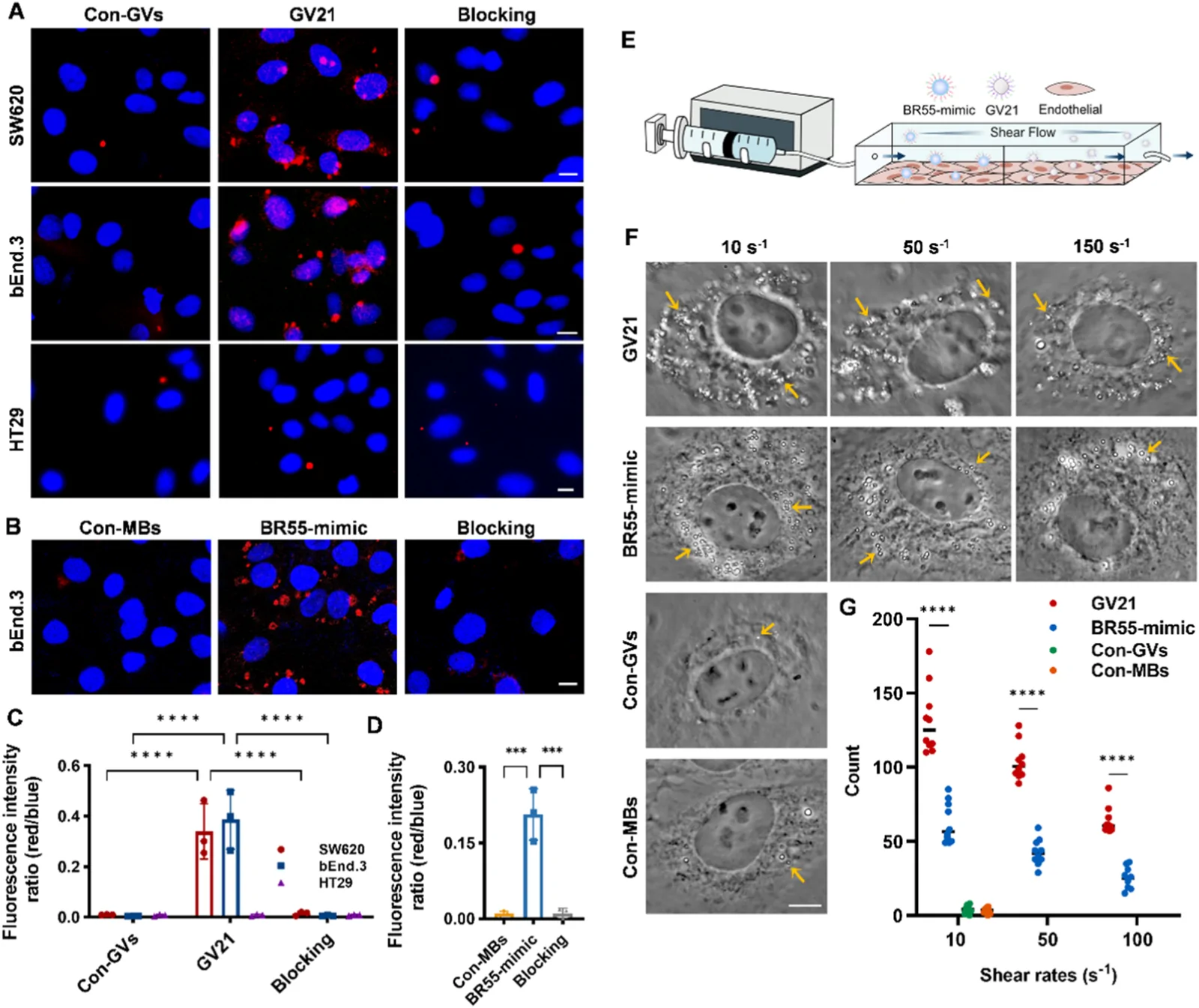
**In vitro adhesion ability of GV21 and BR55-mimic.** (A) Representative fluorescent images of Con-GVs or GV21 binding with SW620, bEnd.3, and HT29 cells. (B) Representative fluorescent images of Con-MBs or BR55-mimic binding with bEnd.3 cells. (C) Quantitative analysis of (A). (D) Quantitative analysis of (B). Free VEGFR2 nanobody (V21) was used to pre-block VEGFR2 receptor. Scale bar = 10 μm. Quantitative analyses in (C) and (D) are presented as mean ± SD (n = 3). (E) Schematic of the flow chamber system used to assess adhesion of GV21 or BR55-mimic to endothelial cells under shear rates. (F) Representative images showing adhesion of Con-GVs, GV21, Con-MBs, and BR55-mimic to endothelial cells under different shear rates in the flow chamber system. Yellow arrows indicate adherent particles. Scale bar = 5 μm. (G) Quantification of adhered particles from (F), n = 10. ***P < 0.001, ****P < 0.0001.

Fluorescence imaging demonstrated that GV21 exhibited significantly higher binding to SW620 and bEnd.3 cells compared with Con-GVs, whereas both GV21 and Con-GVs showed minimal binding to HT29 cells. Notably, pre-blocking with free V21 nanobody markedly reduced GV21 attachment to these VEGFR2-positive cells (Fig. 3A). Similarly, BR55-mimic displayed stronger binding to bEnd.3 cells than Con-MBs, which was substantially inhibited by pre-blocking with V21 nanobody (Fig. 3B). Quantitative analysis revealed that the fluorescence signal intensity ratio (red/blue) of SW620 and bEnd.3 cells in the GV21 groups was approximately 30- and 40-fold higher than in the Con-GVs groups, respectively (*P* < 0.001), with no significant difference observed for HT29 cells (*P* = 0.99, Fig. 3C). The signal intensity ratio of bEnd.3 cells in the BR55-mimic group was 20-fold higher than in the Con-MBs group (*P* < 0.001, Fig. 3D).

Fig. 3E shows a schematic of the flow chamber cell attachment assay. Obviously, GV21 adhered to each bEnd.3 cell at significantly higher numbers than BR55-mimic under shear rates of 10, 50, and 150 s^−1^ (131.47 ± 22.73 vs. 62.35 ± 13.73, 104.22 ± 11.75 vs. 42.73 ± 8.75, and 63.94 ± 9.01 vs. 25.45 ± 7.16, respectively, *P* < 0.001 per group, Fig. 3F and G). In contrast, Con-GVs and Con-MBs showed minimal adhesion even at the lowest shear rates. The number of adherent particles for both GV21 and BR55-mimic decreased as shear rates increased, with GV21 consistently exhibiting higher adhesion than BR55-mimic at all shear rates (Fig. 3F and G), suggesting that the smaller GVs are less prone to detachment under flow.

### 3.4 In vivo USMI of tumor cell-associated VEGFR2

**Fig. 4:**
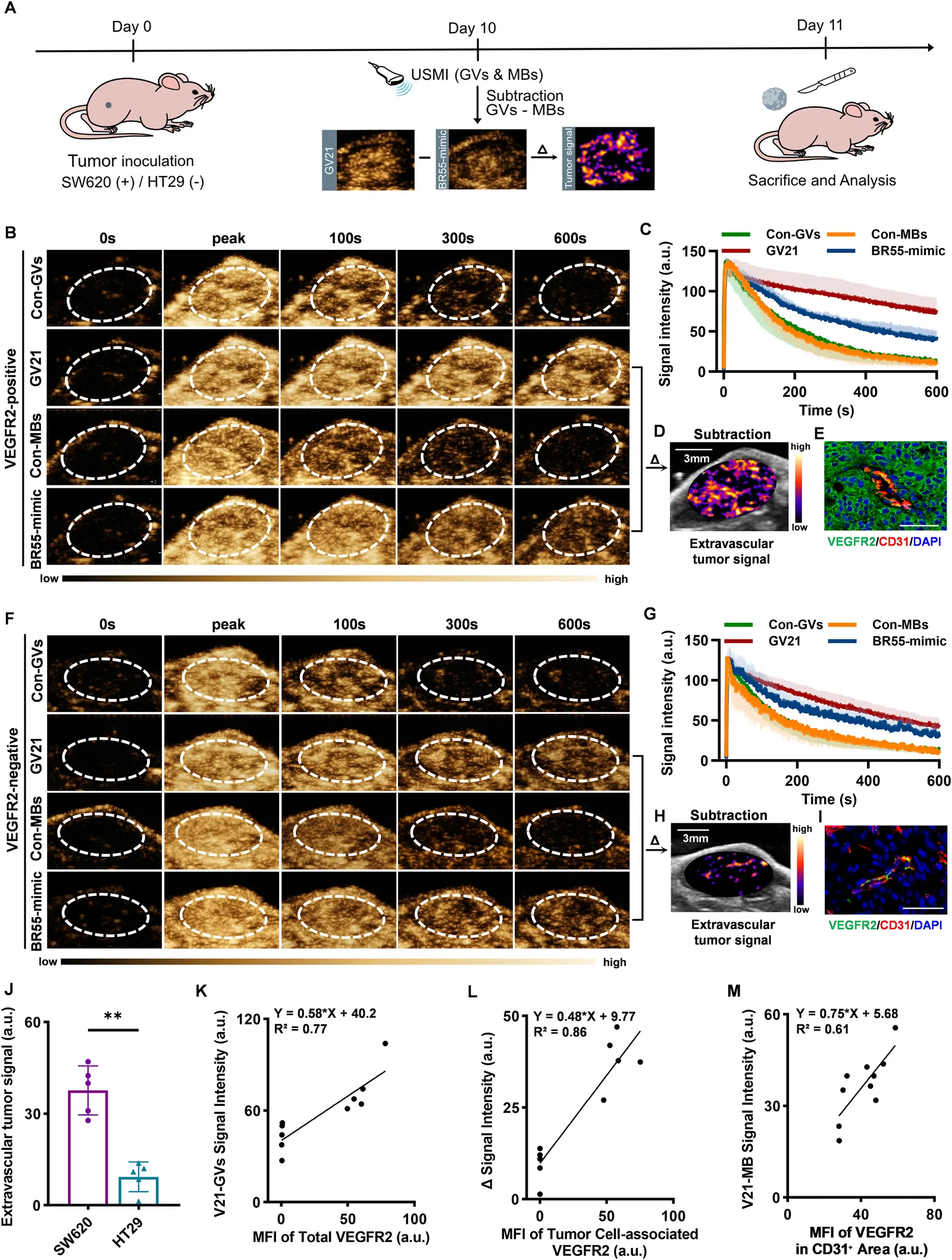
**USMI of GV21 and BR55-mimic for detecting VEGFR2 expression.** (A) Schematic timeline of tumor modeling, USMI acquisition, and signal subtraction in mice bearing tumors derived from VEGFR2-positive (SW620) or VEGFR2-negative (HT29) tumor cells. Representative ultrasound molecular contrast images (B, F) and corresponding time-intensity curves (C, G) of SW620 (B, C) and HT29 (F, G) tumors after injection of Con-GVs, GV21, Con-MBs, and BR55-mimic. (D, H) Subtraction images generated by subtracting the BR55-mimic signals from the GV21 signals at 10 min post-injection in SW620 (D) and HT29 (H) tumors. (E, I) Immunofluorescence staining of CD31 (red), VEGFR2 (green), and nuclei (blue) in SW620 (E) and HT29 (I) tumors. Scale bar: 50 μm. (J) Quantification of subtracted signals in (D) and (H). (K) Correlation between GV21 signal intensity at 10 min post-injection and the mean fluorescence intensity (MFI) of total VEGFR2. (L) Correlation between the subtracted signals (GV21 − BR55-mimic) and the MFI of tumor cell-associated VEGFR2. (M) Correlation between BR55-mimic signal intensity and the MFI of VEGFR2 within CD31-positive regions. Data are presented as mean ± SD (n = 5 per group). **P < 0.01.

USMI was performed in tumors derived from VEGFR2-positive (SW620) or VEGFR2-negative (HT29) tumor cells (n = 5 per group). The timeline of tumor implantation and imaging strategy is shown in Fig. 4A. Obviously, GV21 generated significantly stronger and more sustained signal enhancement in SW620 tumors compared with Con-GVs (Fig. 4B and C), with signal intensity at 10 min reaching 74.27 ± 17.31 vs. 13.11 ± 5.22 a.u. for Con-GVs (*P* < 0.001, Fig. S6A). Similarly, BR55-mimic produced stronger signals compared with Con-MBs (41.53 ± 9.06 vs. 10.91 ± 7.34 a.u., *P* = 0.002, Fig. 4B, C and Fig. S6A). Notably, GV21 generated stronger signals than BR55-mimic, probably attributing to GV21's binding to both tumor vascular endothelial cells and VEGFR2-positive tumor cells (*P* = 0.001, Fig. 4B, C and Fig. S6A). Subtraction of the BR55-mimic signals from the GV21 signals at 10 min enabled detection of tumor cell-associated VEGFR2 expression (Fig. 4D). Immunofluorescence staining further confirmed that VEGFR2 was expressed not only on tumor vasculature but also strongly on SW620 tumor cells (Fig. 4E), supporting the notion that the differential USMI signal from subtraction of the BR55-mimic signals from the GV21 signals reflects VEGFR2 expression levels of tumor cells.

In the tumors derived from VEGFR2-negative HT29 cells, GV21 produced significantly higher signal intensity than Con-GVs at 10 min (42.14 ± 10.05 vs. 13.01 ± 4.62 a.u., *P* < 0.001; Fig. 4F, G and Fig. S6B). Similarly, BR55-mimic generated stronger signals than Con-MBs (31.95 ± 10.47 vs. 10.12 ± 2.51 a.u., *P* = 0.002; Fig. 4F, G and Fig. S6B). However, no significant signal difference was observed between GV21 and BR55-mimic in HT29 tumors (*P* = 0.20; Fig. 4F, G and Fig. S6B). Immunofluorescence further confirmed the absence of VEGFR2 expression on HT29 tumor cells (Fig. 4I). Subtraction of the BR55-mimic signals from the GV21 signals at 10 min only yielded minimal tumor cell-associated signals (Fig. 4H). Quantitative analysis of the subtraction images revealed that the tumor cell-associated signals in SW620 tumors were significantly higher than that in HT29 tumors (37.65 ± 7.01 vs. 9.36 ± 4.85 a.u., *P* = 0.008, Fig. 4J).

As shown in Fig. S7, the MFI of total VEGFR2 (60.76 ± 10.74 vs. 0.65 ± 0.23, *P* < 0.001) and tumor cell-associated VEGFR2 (58.46 ± 10.41 vs. 0.07 ± 0.03, *P* < 0.001) were significantly higher in SW620 compared with HT29 tumors, whereas VEGFR2 expression within CD31-positive regions showed no significant difference (43.25 ± 13.55 vs. 39.39 ± 8.71, *P* = 0.88). In addition, a strong linear correlation (*R*^2^ = 0.77, *P* < 0.001) was observed between the signal intensity of GV21 at 10 min post-injection and the total VEGFR2 expression in tumors (Fig. 4K). Further analysis demonstrated that the subtracted signal (GV21 minus BR55-mimic) was strongly correlated with tumor cell-associated VEGFR2 expression (*R*^2^ = 0.86, *P* < 0.001, Fig. 4L), while BR55-mimic signals correlated with VEGFR2 expression within CD31-positive regions (*R*^2^ = 0.61, *P* = 0.008, Fig. 4M).

To examine the particle distribution in the tumors, AF647-labeled Con-MBs, BR55-mimic, Con-GVs, and GV21 were injected into mice bearing SW620 or HT29 tumors, and tumors were harvested 10 min post-injection for fluorescence imaging (Fig. S8). In both tumor models, minimal fluorescence signals were observed for Con-MBs and Con-GVs, while BR55-mimic were mainly confined to the vascular endothelium. In contrast, GV21 were detected not only along the tumor vasculature but also in extravascular tumor regions in SW620 tumors, whereas only limited extravascular signals were observed in HT29 tumors. These findings further support that the additional USMI signals detected by GV21 arise from tumor cell-associated VEGFR2.

### 3.5 USMI monitoring of apatinib treatment

**Fig. 5:**
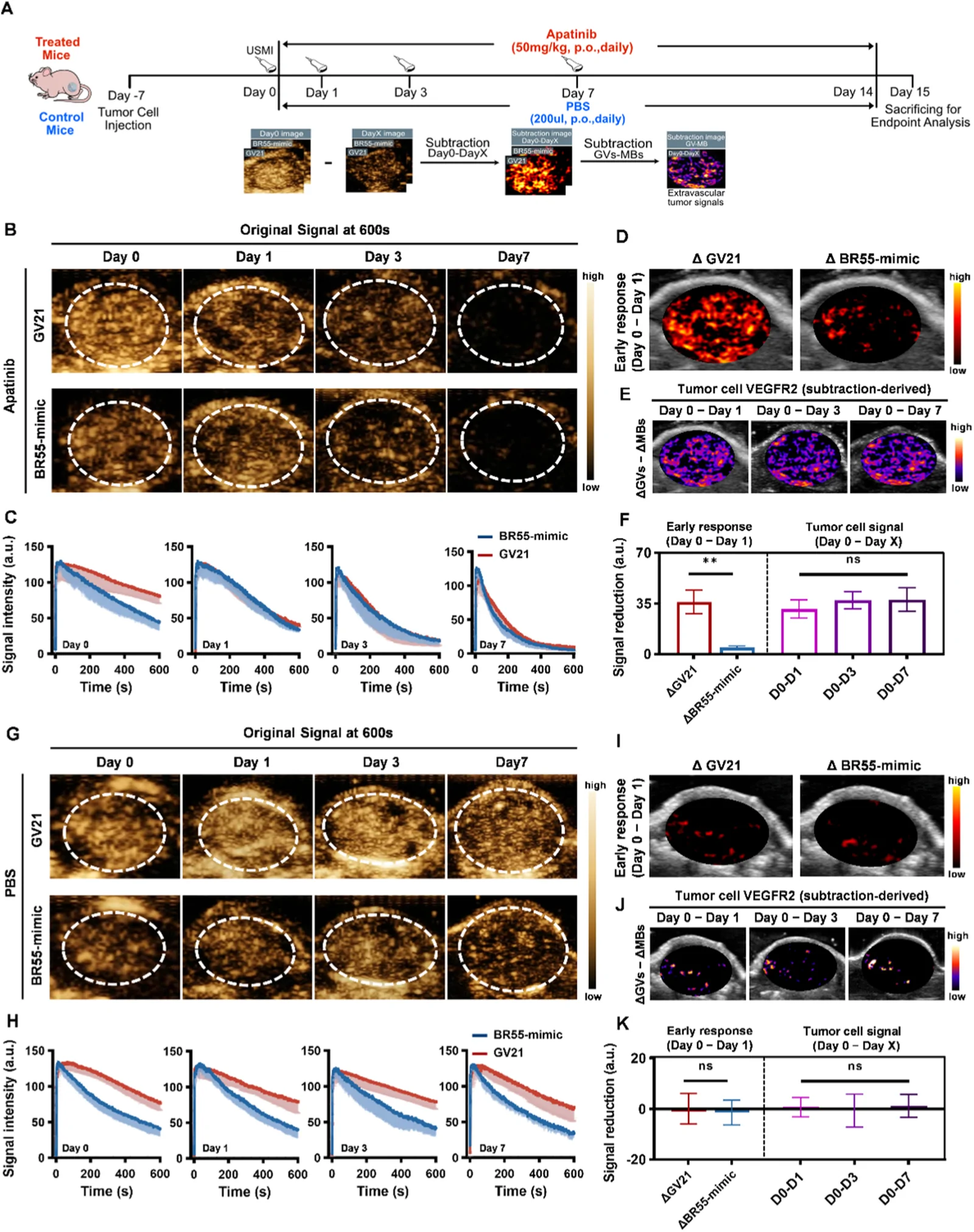
**Longitudinal monitoring of apatinib treatment response using GV21 and BR55-mimic.** (A) Timeline of longitudinal USMI, apatinib or PBS treatment, and signal subtraction in tumors derived from VEGFR2-positive tumor (SW620) cells. Representative USMI images (B, G) and corresponding time-intensity curves (C, H) of the apatinib-treated group (B, C) and PBS group (G, H) tumors after injection of GV21 and BR55-mimic at different time points. Early signal reduction between day 0 and day 1 in the treatment group (D) and PBS group (I). Subtraction images generated by calculating ΔGVs − ΔMBs (signal change from day 0 to each time point) at 10 min post-injection in the treatment group (E) and PBS group (J). (F, K) Quantification of subtracted signals in treatment group (D, E) and PBS (I, J) group, respectively. Data are presented as mean ± SD (n = 5 per group). (**P < 0.01, ns = nonsignificant).

The timeline of tumor treatment and USMI for tumors derived from VEGFR2-positive SW620 cells is illustrated in Fig. 5A. No significant difference in tumor volume was observed 24 h after apatinib treatment, GV21 signal intensity of tumor on day 1 was significantly lower when compared with day 0 (39.60 ± 3.69 vs. 80.63 ± 10.59 a.u., *P* < 0.001; Fig. S9A), as shown in the representative USMI images (Fig. 5B) and the corresponding time-intensity curves (Fig. 5C). In contrast, the BR55-mimic showed only a modest signal decrease that did not reach statistical significance (33.70 ± 4.41 vs. 44.60 ± 11.03 a.u., *P* = 0.097, Fig. 5B, C and Fig. S9A). Both GV21 and BR55-mimic signals further decreased on subsequent time points and were nearly undetectable by day 7 (both *P* < 0.001; Fig. 5B, C and Fig. S9A).

To quantify early treatment response, contrast signal changes relative to baseline (day 0) were calculated (Fig. 5D). On day 1, the tumor-reduced signal from GV21 (ΔGV21) was significantly greater than that from BR55-mimic (ΔBR55-mimic) (36.14 ± 8.01 vs. 4.66 ± 0.96 a.u., *P* = 0.008, Fig. 5D and F). Additional temporal analyses across later time points using baseline subtraction (day 0 − day X) are provided for the apatinib-treated group in Fig. S10, showing progressive signal reduction over time. The signal subtraction of ΔBR55-mimic from ΔGV21 in the same tumor section reflected the extravascular tumor cell-associated VEGFR2 expression reduction, showing comparable levels after apatinib treatment across days 1, 3, and 7 (all *P* > 0.05, Fig. 5E and F), indicative of the lasting effect of the drug for at least 7 days. Representative USMI images at 10 min and the corresponding time-intensity curves for the PBS control group are shown in Fig. 5G and H. In contrast to the apatinib-treated group, the USMI signals of both GV21 and BR55-mimic remained stable over time, with no significant changes observed on day 1, day 3, or day 7 compared with day 0 (all *P* > 0.05, Fig. 5G–K and Fig. S9B). Consistently, baseline-subtracted signals (day 0 − day X) in the PBS group showed no evident changes across time points (all *P* > 0.05, Fig. S11). To evaluate the robustness of the subtraction-based analysis, an alternative calculation approach was applied. As shown in Fig. S12A–D, the changes derived from baseline subtraction of the GVs – MBs signals (day 0 – day 1, day 0 – day 3, and day 0 – day 7) were comparable to those obtained using the ΔGV21 – ΔBR55-mimic method (all *P* > 0.05). These results indicate that both analytical strategies yield consistent outcomes for quantifying tumor cell-associated VEGFR2 signals during treatment monitoring.

### 3.6 Tumor response and VEGFR2 suppression after apatinib treatment

**Fig. 6:**
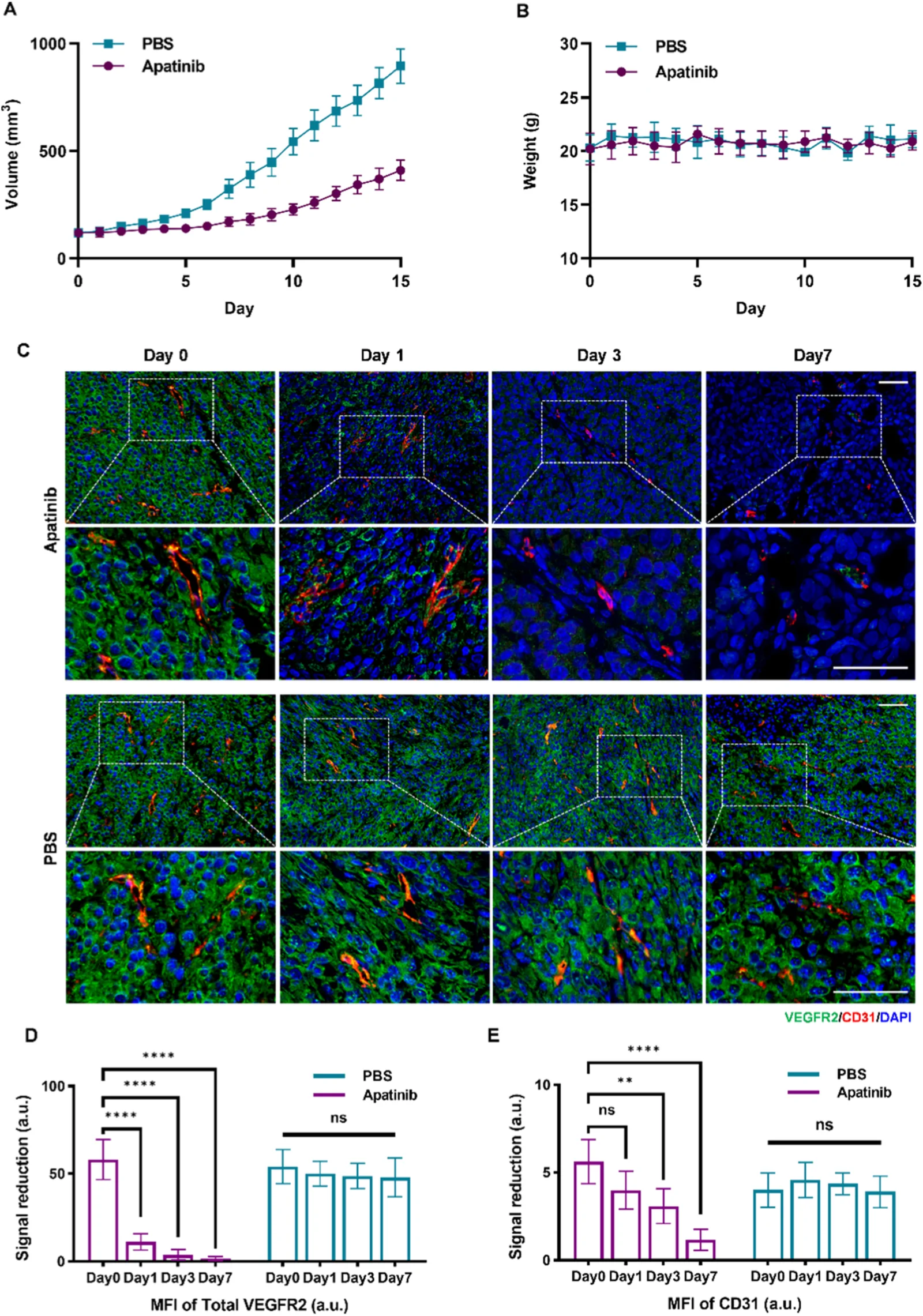
**Therapeutic response to apatinib in SW620 tumor-bearing mice.** (A) Tumor growth curves of mice treated with apatinib or PBS over a 15-day period. Tumor volumes were measured daily. (B) Body weight changes in the apatinib and PBS groups during treatment. (C) Representative immunofluorescence staining of tumor sections from the apatinib and PBS groups at day 0, day 1, day 3, and day 7 after treatment initiation. CD31 is shown in red and VEGFR2 in green. Nuclei were counterstained with DAPI (blue). (D) Quantitative analysis of the MFI of VEGFR2 in tumor sections from the apatinib-treated and PBS-treated groups at different time points. (E) Quantitative analysis of the MFI of CD31 in tumor sections from the apatinib-treated and PBS-treated groups at different time points. Scale bar = 50 μm. Data are presented as mean ± SD (n = 5 per group). (**P < 0.01, ****P < 0.0001, ns = nonsignificant).

Tumor growth curves and body weight changes during apatinib treatment are shown in Fig. 6A and B. Apatinib-treated mice exhibited slower tumor growth compared with PBS controls, with significant tumor growth inhibition observed at later time points, while body weights remained stable in both groups. Immunofluorescence staining of CD31 and VEGFR2 was performed on these tumors collected on days 0, 1, 3, and 7 (Fig. 6C). Following apatinib treatment, MFI of VEGFR2 in tumor cells decreased markedly by day 1 (58.16 ± 11.45 vs. 11.19 ± 4.58, *P* < 0.001, Fig. 6D), whereas vascular VEGFR2 showed only a modest reduction. By day 3, MFI of VEGFR2 further decreased in both tumor cell and vascular compartments, and was minimal by day 7 (both *P* < 0.001, Fig. 6D). In contrast, PBS-treated tumors showed no significant changes in VEGFR2 MFI across all time points (all *P* > 0.05, Fig. 6D). Consistently, CD31 staining demonstrated progressive vascular regression in the apatinib group. No significant change in CD31-positive tumor area was observed on day 1 compared with day 0 (*P* > 0.05). But CD31-positive area was significantly reduced by day 3 (5.61 ± 1.26 vs. 3.08 ± 0.99, *P* = 0.001, Fig. 6E) and further decreased by day 7 (5.61 ± 1.26 vs. 1.16 ± 0.60, *P* < 0.001). By contrast, no obvious changes of CD31 MFI were observed in PBS-treated tumors at all tested time points (all *P* > 0.05). Together, these results validate that GV21-based USMI enables early detection of therapeutic response, which is subsequently reflected in terminal tumor growth inhibition and molecular suppression of VEGFR2.

Western blot analysis further supported the early inhibition of VEGFR2 signaling after apatinib treatment. Compared with pretreatment tumors, tumors collected 24 h after treatment showed reduced VEGFR2 levels and a more pronounced decrease in p-VEGFR2 expression, indicating rapid inhibition of VEGFR2 activation. In addition, decreased p-AKT expression was observed, further supporting suppression of downstream VEGFR2 signaling activity (Fig. S13).

### 3.7 Biosafety of GV21 and BR55-mimic

As shown in Fig. S14A and S14B, SW620 and bEnd.3 cell incubation with GV21 or BR55-mimic for 24 h had no significant impact on cell viability. Hemolysis assays confirmed that both probes did not induce hemolysis at various concentrations (Fig. S15A and B). In vivo, H&E staining of major organs (heart, lung, liver, kidney, spleen) revealed no pathological changes after injection of GV21, BR55-mimic, or PBS (Fig. S16). Furthermore, serum biomarkers of liver (ALT, AST) and kidney (CREA, UREA) function remained within normal ranges after 1 and 7 days post-injection (Fig. S17A and B). Together, these results indicate that GV21 and BR55-mimic are well tolerated and exhibit favorable biosafety.

## 4 Discussion

In this study, we developed GV21 as nanoscale ultrasound contrast agents for USMI of VEGFR2 expression in tumors. Our results showed that GV21 generated higher in vivo VEGFR2 signals than BR55-mimic microbubbles, potentially reflecting their ability to interrogate VEGFR2 expression in both vascular endothelium and extravascular tumor compartments. A subtraction-based imaging strategy further enabled dynamic evaluation of changes in both vascular and tumor cell-associated VEGFR2 expression during anti-VEGFR2 apatinib treatment. Using this approach, GV21-based USMI provides more comprehensive characterization of VEGFR2-associated signals within tumors and improves the detection of early responses to VEGFR2-targeted therapy.

Targeted microbubbles, particularly VEGFR2-targeted agents such as BR55, have been widely investigated for USMI of tumor angiogenesis and have demonstrated encouraging performance in both preclinical and early clinical studies [3,4,8,9,26]. However, their intravascular localization limits detection of molecular targets beyond the vascular endothelium. In contrast, the nanoscale size of GVs may facilitate access to tumor interstitial regions and enable detection of VEGFR2-associated signals beyond the vascular endothelium [27]. Nevertheless, GV21 and BR55-mimic differ in several physicochemical properties, including particle size, concentration, acoustic behavior, and ligand density. Due to their larger size and distinct acoustic properties, individual BR55-mimic microbubbles generally generate stronger ultrasound signals than individual GV21 particles [28,29]. To achieve comparable contrast enhancement in tumor imaging experiments, the administered doses were adjusted accordingly: a higher particle concentration of GV21 was used to compensate for the weaker per-particle signal, whereas fewer BR55-mimic particles were required due to their stronger acoustic response. Although individual microbubbles possess higher ligand density, the substantially greater number of GV21 particles administered resulted in a higher estimated total amount of V21 ligands per injection. Therefore, the enhanced VEGFR2 molecular imaging performance of GV21 may be attributed not only to its nanoscale size and potential extravascular accessibility, but also to the increased number of targeting particles delivered, collectively contributing to improved detection of VEGFR2-associated signals.

To evaluate the functional relevance of GV21-based USMI, apatinib, a selective small-molecule VEGFR2 tyrosine kinase inhibitor, was selected as a preclinical therapeutic model [30,31]. Since VEGFR2 inhibition affects both tumor angiogenesis and VEGFR2-associated tumor cell signaling pathways [32,33], apatinib serves an appropriate model to assess treatment-induced molecular changes detected by different ultrasound contrast agents. During treatment, GV21-based USMI revealed rapid decreases in VEGFR2-associated signals, whereas BR55-mimic-based USMI primarily reflected vascular VEGFR2 changes due to its intravascular localization. Furthermore, subtracting BR55-mimic signals from GV21 signals within the same tumor region enabled estimation of the extravascular VEGFR2-associated signal contribution, providing a strategy to improve the biological interpretation of USMI signals. These findings suggest the potential of nanoscale ultrasound contrast agents for more comprehensive monitoring of therapeutic responses.

Consistent with these findings, GV21 signals exhibited a marked decline as early as 24 h after apatinib administration, whereas BR55-mimic signals showed minimal changes at this early time point (Fig. 5B and C). This difference may reflect the distinct cellular compartments sampled by the two contrast agents. Previous studies have demonstrated that pharmacological inhibition of VEGFR2 by apatinib rapidly inhibits receptor phosphorylation and downstream signaling pathways, leading to early alterations in tumor cell-associated VEGFR2 activity [30,31]. In contrast, VEGFR2 expressed on vascular endothelial cells represents a relatively stable receptor pool during the early phase of anti-angiogenic therapy, and structural changes in tumor vasculature generally require several days to become evident [34]. Therefore, the earlier decline in GV21 signals may reflect changes in VEGFR2-associated signals in both vascular and extravascular tumor regions, whereas BR55-mimic predominantly detects intravascular VEGFR2 changes. These results suggest that GV21 may serve as more sensitive molecular probes for evaluating early therapeutic responses by enabling assessment of VEGFR2-associated signals beyond the vascular compartment.

Despite these promising findings, several limitations of this study should be acknowledged. First, the subtraction-based strategy provides an indirect approximation of tumor cell-associated VEGFR2 signals, and further validation using complementary histological or molecular approaches would strengthen the interpretation of these imaging results. Second, this study was conducted in a limited number of tumor models, and future investigations involving additional tumor types are needed to determine the general applicability of this approach. Third, although GVs have shown promising imaging performance in preclinical studies, further work is still required before clinical translation. For example, scalable manufacturing and batch-to-batch consistency of biosynthetic GVs need to be further optimized, especially for large-scale isolation and purification with clinically approved reagents under mild conditions. In addition, comprehensive evaluation of long-term pharmacokinetics, biosafety, and immunogenicity need to be evaluated before clinical application [35,36]. Moreover, although BR55 has advanced to clinical evaluation, with first-in-human studies demonstrating its safety and feasibility for molecular ultrasound imaging [9,10], GV21 remains at the preclinical stage. Future studies should further assess the clinical feasibility of GV21 through direct comparison with BR55 in clinically relevant models, together with the development of standardized manufacturing and quality-control procedures to facilitate clinical translation.

In summary, our findings suggest that GV21 enables sensitive and more comprehensive USMI of VEGFR2 expression in tumors by detecting both vascular and tumor cell-associated VEGFR2 expression. When combined with VEGFR2-targeted therapies such as apatinib, this approach provides a promising imaging strategy for early and noninvasive monitoring of therapeutic responses, potentially improving the assessment of therapeutic efficacy in preclinical and clinical settings.

## CRediT authorship contribution statement

**Xiaoxin Liang:** Writing – review & editing, Writing – original draft, Project administration, Methodology, Investigation, Data curation, Conceptualization. **Shilin Lu:** Writing – review & editing, Writing – original draft, Project administration, Data curation, Conceptualization. **Liang Yang:** Writing – review & editing, Project administration. **Jianhua Zhou:** Writing – review & editing, Supervision, Resources, Methodology, Investigation, Funding acquisition, Conceptualization. **Fei Yan:** Writing – review & editing, Visualization, Validation, Supervision, Resources, Methodology, Investigation, Funding acquisition, Data curation, Conceptualization.

## Funding

The authors gratefully acknowledge the support of the 10.13039/501100012166National Key R&D Program of China (2025YFA0922100), the Shenzhen Medical Research Fund (B2402006), the Leading Talents of the Guangdong Special Support Program (2024TX08A098), the Shenzhen Synthetic Bio-Manufacturing Pilot-scale Base Project (ZXSJD20240708092506009), and the 10.13039/501100003453Natural Science Foundation of Guangdong Province in China (2023B1515120007).

## Declaration of competing interests

The authors declare that they have no known competing financial interests or personal relationships that could have appeared to influence the work reported in this paper.

## Data Availability

No data was used for the research described in the article.
