# Supplementary material for: GV21 enables ultrasound molecular imaging of tumor cell-associated VEGFR2 for early therapy assessment
Source: Mater Today Bio. 2026 Sep 8;40:103659. doi: 10.1016/j.mtbio.2026.103659 (PMC13586588; doi:10.1016/j.mtbio.2026.103659)
Supplement: Supplementary file 1 [file mmc1.docx]

 **Figure S1.** Schematic representation of the preparation of VEGFR2-targeted gas vesicles (GV21) and VEGFR2-targeted microbubbles (BR55-mimic).

**Figure S2. MALDI-TOF mass spectrum of the purified V21 nanobody.** The spectrum shows a predominant [M+H]^+^ ion at m/z 15,598.56, corresponding to the measured molecular mass of V21 and consistent with the expected molecular mass (~15 kDa). The additional peak at m/z 7,795.38 is assigned to the doubly charged [M+2H]^2+^ ion of V21.

**Figure S3.** Western blot analysis of VEGFR2 and β-actin expression in different cell lines.

**Figure S4.** Immunofluorescence staining (A) and flow cytometry (B) of VEGFR2 of bEnd.3, SW620 and HT29 cells using a commercial full-length anti-VEGFR2 antibody. Scale bar: 10 µm.

**Figure S5.** Flow cytometry analysis of VEGFR2 expression on bEnd.3, SW620, and HT29 cells using the V21 nanobody.

**Figure S6.** Quantification of USMI signal intensity at 10 min post-injection of Con-GVs, GV21, Con-MBs, or BR55-mimic in tumors derived from SW620 (A) and HT29 (B) cells, n = 5 per group. The data showed as mean ± SD. (***P* < 0.01, ****P* < 0.001, *****P* < 0.0001, ns = nonsignificant).

**Figure S7.** Quantification analysis of the mean fluorescence intensity of total VEGFR2, tumor cell-associated VEGFR2, and VEGFR2 expression within CD31-positive regions from SW620 tumors and HT29 tumors, n = 5 per group. The data showed as mean ± SD. (*****P* < 0.0001, ns = nonsignificant).

**Figure S8**. Representative fluorescence images of tumor sections from mice bearing SW620 (top) and HT29 (bottom) tumors after intravenous injection of AF647-labeled Con-MBs, BR55-mimic, Con-GVs, or GV21. Tumor vasculature was stained with CD31 (green), nuclei with DAPI (blue), and contrast agents are shown in red. Scale bar: 50 μm.

**Figure S9**. Quantification of USMI signal intensity at 10 min post-injection of Con-GVs, GV21, Con-MBs, or BR55-mimic in tumors derived from aptinib (A) and PBS (B) groups, n = 5 per group. The data showed as mean ± SD. (****P* < 0.001, *****P* < 0.0001, ns = nonsignificant).

**Figure S10**. (A) Subtraction images generated by calculating ΔGVs − ΔMBs (signal change from day 0 to each time point) at 10 min post-injection in the treatment group. (B) Quantification of subtraction signals from (A).

**Figure S11**. (A) Subtraction images generated by calculating ΔGVs − ΔMBs (signal change from day 0 to each time point) at 10 min post-injection in the PBS group. (B) Quantification of subtraction signals from (A).

**Figure S12.** (A) Representative baseline subtraction images (GV21 − BR55-mimic) at day 0, 1, 3, and 7 in the apatinib and PBS groups. (B) Quantification of the baseline subtraction signals shown in (A), n = 5 per group. (C) Representative temporal subtraction images Δ(GV21 − BR55-mimic) calculated from day 0 to day X. (D) Quantification of the temporal subtraction signals shown in (C), n = 5 per group. The data showed as mean ± SD. (****P* < 0.001, *****P* < 0.0001, ns = nonsignificant).

**Figure S13.** Representative western blot images of VEGFR2, p-VEGFR2, p-AKT and β-actin levels in tumor tissues collected before and 24 h after apatinib administration.

**Figure S14.** (A) Viability of SW620 cells and bEnd.3 cells after treatment with GV21 at of different concentrations for 24 h, n = 3. (B) Viability of bEnd.3 cells after treatment with BR55-mimic at of different concentrations for 24 h, n = 3.

**Figure S15**. Hemolysis of red blood cells in the presence of GV21 (A) or BR55-mimic (B) with various concentrations (n = 3). PBS and distilled water were used as negative and positive controls, respectively.

**Figure S16**. Representative H&E images of major organs (heart, liver, spleen, lung and kidney) from the mice received with PBS, GV21 or BR55-mimic after 7 days. Scale bars: 100 µm.

**Figure S17**. 1 day and 7 days after injection of 100 μL of PBS, GV21 or BR55-mimic, serum biochemical values, including alanine aminotransferase (ALT), aspartate aminotransferase (AST), UREA and creatinine (CREA), n = 3. The data showed as mean ± SD.
